# How does the work-life balance impact stress on primary healthcare workers during the COVID-19 pandemic?

**DOI:** 10.1186/s12913-023-09677-0

**Published:** 2023-07-05

**Authors:** Nuzulul Kusuma Putri, M. Karomah Nastiti Melania, Sia Mawan Yulia Fatmawati, Yin Cheng Lim

**Affiliations:** 1 Health Policy and Administration Department, Faculty of Public Health, Universitas Airlangga, Surabaya, Indonesia; 2 The Airlangga Centre for Health Policy, Surabaya, Indonesia; 3 Kediri Regency Health Office, Kediri, Indonesia; 4 Department of Social and Preventive Medicine, Faculty of Medicine, University of Malaya, Kuala Lumpur, Malaysia

**Keywords:** Mental health, Primary healthcare, COVID-19 pandemic, Work-life balance, Stress

## Abstract

**Background:**

Most studies in advanced care settings reported that the increasing workload increases the work-life imbalance and harms the mental health of health workers. The COVID-19 Pandemic's tracing, testing, treatment, and mass vaccination also have multiplied the primary healthcare workers' workload. Nevertheless, studies on primary care workers are scarce. This study aimed to investigate how the COVID-19-related work-life balance impact stress on primary healthcare workers in the third years of the pandemic.

**Methods:**

The study was a cross-sectional, web-based survey conducted on primary healthcare workers in Kediri Regency, Indonesia, with the highest Omicron case surge worldwide. It was conducted right after the surge between July and August 2022, the third year of the COVID-19 pandemic hit Indonesia. Under coordination with the local government health officials, primary healthcare workers were invited to participate in an online survey. The respondents were asked to evaluate their sociodemography, work conditions, personal life, and perceived stress (using the Perceived Stress Scale) during the pandemic. Their work-life balance was evaluated using the Work/Non-work Interference and Enhancement Scale. We used several hierarchical linear regression models to determine which variables contribute to work stress among primary healthcare workers.

**Results:**

Sociodemographic characteristics, including gender, age, marital status, years of professional experience, and educational level, were not significantly associated with stress levels among our respondents. Separately, work conditions and personal life variables did not associate with stress levels. However, primary healthcare workers' work and personal lives interfere with each other during the pandemic and are associated with their higher stress.

**Conclusion:**

During the pandemic, the work life of primary health workers interferes with their personal life more than the interference of personal life on their work life. At the same time, the work life's enhancement on the personal life and vice versa were lower than its interference. Those conditions are associated with higher perceived stress of primary health workers.

**Supplementary Information:**

The online version contains supplementary material available at 10.1186/s12913-023-09677-0.

## Background

Health workers reportedly experienced depression, anxiety, and stress during the COVID-19 pandemic [1]. Not only already anxious due to their vulnerability to transmitted COVID-19 from their patients, but health workers were also stigmatized related to COVID-19 at the beginning of the pandemic [2]. Health workers' poor quality of work-life due to the COVID-19 pandemic reportedly harms their physical and psychological wellbeing, leading to higher stress [3]. Working in high-risk COVID-19 transmission works developed a perceived threat of COVID-19, combined with work overload, limited resources for COVID-19 handling, and poor social support at work manifested in a higher stress level [4]. Years after the pandemic started, health workers were consistently experiencing stress and job burnout even after the peak of the COVID-19 pandemic, and the lockdown policy was lifted [5]. The risk of mental ill health due to the COVID-19 pandemic has been increasing worldwide, demanding more studies adopting psychological instruments that can be used in healthcare settings [6, 7].

Primary healthcare workers are health workers with a crucial role in every COVID-19 control measure. In Indonesia, with more than 6.6 million COVID-19 cases since the pandemic's beginning until November 2022, primary healthcare workers are central in the COVID-19 tracing, testing, and treatment. Tracing and testing are all the primary health workers' responsibilities. While only 20% of COVID-19 patients are treated in hospitals, the remaining 80% are self-quarantined and become the responsibility of primary healthcare facilities to monitor [8]. It burdens a high workload for primary health workers, leading to burnout and stressful work life [8, 9]. The workload has multiplied since COVID-19 mass vaccination are also assigned to primary healthcare facilities.

Worldwide, primary healthcare workers are reportedly experiencing stressful work conditions during the COVID-19 pandemic. In a low-income country, Malawi, the high workload of primary healthcare facilities during the COVID-19 pandemic was worsened by a lack of resources (human and material) inadequacy, resulting in fatigue and high stress among its primary healthcare workers [10]. The same stressful work conditions also happen among primary healthcare facilities in high-income countries. Due to multiple tasks related to COVID-19, primary health workers in Saudi Arabia reportedly must deal with challenging role conflict in their job and limited social support [11]. It also changed Sweden's work organization and routine in primary healthcare facilities, pushing its workers to adapt abruptly to stressful conditions [12]. In Malaysia and Spain, primary health workers feared handling COVID-19 even after years of the pandemic, initiating continuous psychological distress [13, 14]. Even the medical liability of the population to be COVID-19 vaccinated has brought legal repercussions for healthcare workers in the European Union, increasing their stress and burnout [15]. This fear is also combined with their worries about any risk of transmitting the disease to their family members [14].

There is acceptable evidence of how the COVID-19 pandemic has impacted primary health workers' work and personal lives. Work-life balance is crucial in keeping health workers satisfied with their work conditions to minimize stress and maintain harmony in their workplace [16]. Work-life balance among professionals, including health professionals, is the key to better efficiency [17]. However, most studies only investigated how the pandemic impacted health workers' work-life balance and work stress in the hospital setting [16, 18, 19]. Unfortunately, limited studies investigated how the pandemic impacted primary healthcare workers in the community setting. Hence, this study aims to verify whether work-life imbalance due to COVID-19 contributes to working stress among primary healthcare workers. Our study contributes to understanding how the COVID-19 pandemic impacts the stress level among primary health workers who work daily in a community setting.

## Methods

### Design

It was a cross-sectional study conducted in Kediri Regency, Indonesia. The study population was all healthcare workers in primary healthcare facilities in the regency. The responsibilities of healthcare workers in fighting the pandemic include crucial steps such as COVID-19 tracing, testing, and vaccination, which demand significant resources of health workers and can interfere with their personal lives.Data from Regency Health Office showed 1,260 primary healthcare workers in 37 primary healthcare facilities in 2021. The sample needed for this study was calculated using proportional sampling, considering a statistical significance level of 0.05, which was 304 observations. However, the final sample comprised 308 primary healthcare workers.

### Data collection

An online questionnaire using the SurveyMonkey platform was used to collect the data. Before the survey, an online meeting with all managers of primary healthcare facilities was conducted to explain the study's objectives. We cooperated with Regency Health Office to distribute the survey link to all health workers in primary healthcare facilities. Data collection started on July 26 and was completed at the end of August 2022, right after the surge of the Omicron case in Indonesia. The first Omicron wave ended in early May with only 106 new cases in a day, and it rose again to the highest record of new cases on July 28, with 6,353 new cases in one day. All the participants were volunteers. Regency Health Office sent weekly reminders to all primary healthcare facilities to increase the response rate. Informed consent was the first question in the survey, and only participants who consented could continue completing the online survey.

### Variables and instruments

#### Sociodemography

Several variables related to COVID-19's mental health impacts on health workers were reported. Those variables were gender, age, marital status, years of professional experience, and educational level.

#### Work condition

To capture determinants of work conditions, we asked the healthcare workers to provide information about their occupation, the number of hours spent working in primary healthcare facilities per day, extra tasks as assigned, extra tasks related to COVID-19 handling, and side hustles outside their main job as health workers in primary healthcare facilities.

#### Personal life

Variables we used to describe personal life were related to the health workers' domestic workload at home. First, we asked whether they have daily responsibilities for any dependents at home, including elderly and/or under-five children. Second, we asked them whether they got a domestic servant to help finish their domestic work at home.

#### Work-life balance

The balance between the health worker's personal and working life was evaluated using the Work/Non-work Interference and Enhancement Scale by Fisher et al. (2009) [20]. It captured four work-life balance conditions, including the condition of whether their work interference with personal life (WIPL), personal life interference with work (PLIW), work enhancement of personal life (WEPL), and personal life enhancement of work (PLEW).

Role interference is processed from simultaneous pressure where compliance on one role makes compliance to another more difficult [21]. Sieber (1974) reported that managing multiple roles could not only result in unfavorable conditions; it also initiates enhancement in fulfilling other roles [22]. Based on Fisher et al.'s Work/Non-work Interference and Enhancement Scale, work-life balance determines job stress and satisfaction. The Work/Non-work Interference and Enhancement scale consisted of 14 questions where WIPL and PLIW have five questions each and WEPL and PLEW has two questions each. Respondents needed to answer with the frequency they had the thoughts presented in each item using a Likert-type scale ranging from 1 (never) to 5 (very often) for WEPL and PLEW, and 5 (never) to 1 (very often) for WIPL and PLIW. The average score of each dimension represented the health workers' work-life balance [23, 24]. Many studies used the same instrument from Fisher et al. (2009) to assess WLB and used its average score to categorize the high/low pattern of each dimension [20, 25] and compare the substantive among those dimensions [26, 27]. Fishers et al. (2009) used the scale's midpoint [3] to categorize each work-life balance dimension [20, 25].

*Work Interference with Personal Life (WIPL),* this dimension refers to the degree to which work interferes with one's personal life. Interference between work on personal life means that their work demands more resources, creating difficulties in fulfilling their personal responsibilities and making them unsatisfied with their job. In this study, we used inverted scales for WIPL to compare its average scores with enhancement dimensions. The increase in WIPL means to be positive for work-life balance. A higher score of WIPL represents a lower conflict of work in someone's personal life and is associated with lower job stress.

*Personal Life Interference with Work (PLIW),* this dimension refers to the degree to which an individual's personal life interferes with his/her work life. Someone experiencing problems in their personal lives can discomfort them during work, creating an uncomfortable work situation. We used inverted scales for PLIW. In this study, a higher score of PLIW represents a lower conflict between personal life in work life. Hence, the increasing score of PLIW is associated with decreased job stress and increased life satisfaction.

*Work Enhancement of Personal Life (WEPL),* this dimension refers to the extent to which a person's personal life enhances individual performance in his/her work life. WEPL indicated how their work life renews resources that are useful in their personal life, making them satisfied with their work and having a higher score of WEPL associated with higher job satisfaction and lower job stress.

*Personal Life Enhancement of Work (PLEW),* this dimension refers to the extent to which a person's work life can improve the quality of an individual's life in his/her personal life. This dimension is positively related to life satisfaction. The increasing score of PLEW increases job satisfaction.

This study interpreted a higher WIPL and PLIW as a condition with less conflict between roles, indicating a more favorable work-life balance. While the high score of WEPL and PLEW showed that managing roles' conflict results in enhancement and is favorable for work-life balance. Those four dimensions were used as separate factors in the analysis, allowing this study to compare which dimensions contribute more to WLB [24, 28]. In our study, Cronbach's alpha coefficients were 0.85 for WIPL, 0.87 for PLIW, 0.82 for WEPL, and 0.88 for PLEW.

#### Perceived stress scale

This scale measured how different situations affect someone's feelings and perceived stress [29]. There were ten questions in this scale where the respondents were asked to choose the best response that represented how often they felt or thought a certain way. The responses were a Likert-type scale ranging from 0 (never) to 4 (very often). The Perceived Stress Score was calculated by averaging all the questions after reverse scores for questions 4, 5, 7, and 8. Individual scores can range from 0 to 4, with higher scores indicating higher perceived stress. Scores ranging from 0–1.3 are considered low stress, 1,4–2.6 as moderate stress, and 2.7–4 as high perceived stress. Cronbach's alpha for the perceived stress scale in this study is 0.79.

**Fig. 1:**
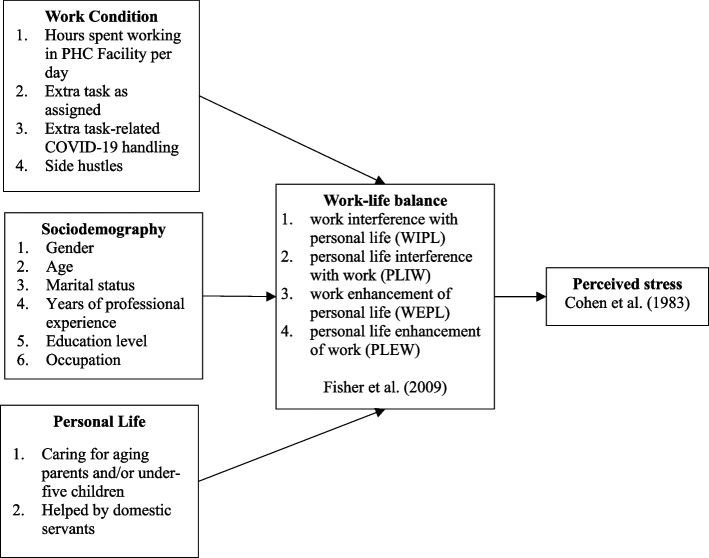
Conceptual diagram

Figure 1 is our conceptual framework which illustrates the variables we studied and the relationships we expect to find between them.

### Statistical analysis

We presented all variables descriptively by providing the frequency and percentage. We provided mean and standard deviation for the continuous variables, including work-life balance and stress score. We used the average score of each dimension to compare which dimensions of work-life or personal life dominantly bring any interference or enhancements toward someone’s work-life balance. We conducted a collinearity test to guarantee no strong correlation between independent variables in the final regression model (the mean of the VIF value is 1.36). We then inspected the correlations among variables using Pearson's correlation (Supplementary Table 1).

We used several hierarchical linear regression models to determine what variables contribute to working stress among primary healthcare workers. We used dummy coding for nominal variables, including gender, marital status, educational status, occupation, extra work assigned, extra task-related COVID-19 handling, and side hustles. We used the perceived stress score as the only outcome in all linear regression models in this study. Our first model's explanatory variables were sociodemography, including gender, age, marital status, years of professional experience, and educational level. We then added work condition variables into the base model to investigate how the health workers' roles in their primary healthcare facilities contribute to their stress. Extra tasks as assigned represented additional workload in their primary healthcare facilities, even though not during the COVID-19 pandemic. We also considered side hustles outside the primary healthcare facility as another workload that potentially brings any role conflict. The extra task-related COVID-19 handling variable represented specific tasks related to the pandemic assigned to the health workers during the pandemic. For this variable, we differentiated the task based on specific COVID-19 pandemic control measures that become the responsibilities of primary healthcare facilities. We added our base model with personal life variables in the third model. The health workers' responsibility to care for their aging parents and/or under-five children and whether a servant helped them finish all their domestic work were used as the personal life variables. Our fourth model was our moderation analysis investigating whether the work-life balance moderates all work and personal life conditions in determining the health workers' stress. Finally, in the fifth model, we tested all explanatory variables jointly on the stress of health workers.

## Results

Three hundred-eight primary healthcare workers representing all primary healthcare facilities in Kediri Regency participated in this study. Table 1 shows that most of our respondents were female (84.7%) and married (84.7%). The distribution of professional experience years likely showed that most of the respondents were in their early career stage since most of them were aged 26–30 years old (24.0%), had less than five years of professional experience (36.7%), and only in their bachelor's education degree (51.6%).

**Table 1:** Sociodemographic, work life balance and perceived stress of primary healthcare workers

**Variables**	**Freq. or mean**	**Percentage (%)**	**Standard deviation**
**Sociodemography**			
**Gender**			
Male	47	15.3%	
Female	261	84.7%	
**Age**			
< 25 years	26	8.4%	
26–30 years	74	24.0%	
31–35 years	41	13.3%	
36–40 years	46	14.9%	
41–45 years	49	15.9%	
46–50 years	35	11.4%	
51–55 years	29	9.4%	
56–60 years	8	2.6%	
**Marrital status**			
Single	34	11.0%	
Widowed/divorced	13	4.2%	
Married	261	84.7%	
**Years of professional experience**			
0–5 years	113	36.7%	
6–10 years	35	11.4%	
11–15 years	54	17.5%	
16–20 years	36	11.7%	
21–25 years	27	8.8%	
26–30 years	30	9.7%	
31–35 years	13	4.2%	
**Educational level**			
Vocation	147	47.7%	
Bachelor	159	51.6%	
Master	2	0.6%	
**Work condition**			
**Occupation**			
Midwife	81	26.3%	
Nurse	68	22.1%	
Sanitarian	29	9.4%	
Physician	15	4.9%	
Pharmacist	15	4.9%	
Nutritionist	15	4.9%	
Dentist	15	4.9%	
Laboratorist	14	4.5%	
Health promotor	14	4.5%	
Other	42	13.6%	
**Hours spent working in primary healthcare facilities per day**			
no more than 4 h	9	2.9%	
4–8 h	293	95.1%	
more than 8 h	6	1.9%	
**Extra task as assigned**			
No	70	22.7%	
Yes	238	77.3%	
**Extra task-related COVID-19 handling**			
Do not have extra task	70	22.7%	
Testing	11	3.6%	
Tracing	29	9.4%	
Treating	7	2.3%	
Vaccination	139	45.1%	
Have extra tasks (related COVID-19) but no specific task assigned	52	16.9%	
**Side hustles**			
No	220	71.4%	
Yes	88	28.6%	
**Personal life condition**			
**Caring for aging parents and/or under-five children**			
No	129	41.9%	
Yes	179	58.1%	
**Helped by domestic servants**			
No	155	50.3%	
Yes	153	49.7%	
**Work interference with personal life (WIPL) score**	3.41		0.86
**Personal life interference with work (PLIW) score**	4.23		0.74
**Work enhancement of personal life (WEPL) score**	3.4		0.85
**Personal life enhancement of work (PLEW) score**	3.74		0.84
**Stress score**	1.53		0.5

Table 1 also describes that most of our respondents were midwives (26.3%) and spent 4–8 h working in primary healthcare facilities (95.1%). However, at least 28.6% of respondents reported having other jobs besides working in their home primary healthcare facilities. Most respondents also reported being assigned extra tasks outside their main occupation task (77.3%). During the COVID-19 Pandemic, most respondents were assigned tasks related to pandemic handling, most of which were assigned to the COVID-19 vaccination program. Even though 22.7% asserted that they did not get any extra task-related COVID-19, 16.9% of respondents claimed that they were loaded to do multiple task-related COVID-19 handling. Regarding the personal life condition of health workers, most respondents reported that they took care of their dependents while working (58.1%). More than half of them also reported that they did their domestic work independently without help from domestic servants (50.3%).

Among the work-life balance score dimensions, the PLIW score was the highest (mean = 4.23; SD = 0.74) and higher than WIPL (mean = 3.41; SD = 0.86). First, it indicated that although both dimensions with enhancement condition were lower than those with interference condition, PLEW (mean = 3.74; SD = 0.84) and WEPL (mean = 3.4; SD = 0.85), personal lives were advantageous for health worker’s work-life balance since their personal lives bring less interference to their work life. Moreover, the PLIW (mean = 4.23; SD = 0.74) and PLEW score (mean = 3.74; SD = 0.84) were relatively high since the scores of PLIW and PLEW in this study are significantly higher than the scale's midpoint [3]. It indicates that their personal life was likely associated with a more beneficial condition on their working life. Second, since the PLIW score was higher than WIPL, it indicated that the personal life interferences on their working life were less than how their working life conflicted with their personal life. In contrast, the WIPL (mean = 3.41; SD = 0.86) and WEPL (mean = 3.4; SD = 0.85) scores were lower than other dimensions, indicating that working life brought interference and less enhancement to the personal lives of health workers. Meanwhile, the perceived stress score was moderate; the mean stress score was 1.53 (SD = 0.5), suggesting relatively moderate-stress levels among primary health workers. The correlation test showed in Supplementary Table 1, as expected, that the score of all work-life balance dimensions at p < 0.001, PLIW (-0.554), PLEW (-0.409), WIPL (-0.475), WEPL (-0.370), were negatively correlated with stress which means those higher scores likely associated with lower stress levels.

Table 2 presents the results of multiple linear regressions. In our first model, we used socioeconomic demography variables, including gender, age, marital status, years of professional experience, and educational level, to seek their propensity resulting in stress. However, we found that those variables were not significantly associated with stress levels among our respondents. We then added work condition variables into this first model to test how work conditions are associated with stress levels. We did not find any significant association with stress in this second model. Our third model replaced work condition variables with personal life condition variables. However, our linear regression also showed no personal life condition variables significantly associated with stress. The results of our second and third models indicated that a single role of health workers, related to work or personal life, was not significantly associated with stress on health workers. Our fourth model supported it, using work-life balance dimensions as a predictor of primary health workers' stress significantly associated with their stress level.

**Table 2:** Predictors of stress among primary health workers

Variables	(1)	(2)	(3)	(4)	(5)
	Model 1	Model 2	Model 3	Model 4	Model 5
Male	-0.00326	0.0168	0.0115	-0.0760	-0.0429
	(0.0808)	(0.0861)	(0.0811)	(0.0601)	(0.0645)
Age	-0.00292	-0.00420	-0.00168	-0.000861	-0.000563
	(0.00342)	(0.00374)	(0.00372)	(0.00254)	(0.00300)
Widowed/divorced	0.0167	0.0124	-0.00869	0.114	0.111
	(0.177)	(0.181)	(0.178)	(0.132)	(0.135)
Married	0.0836	0.0748	0.0618	0.0341	0.0327
	(0.101)	(0.104)	(0.102)	(0.0758)	(0.0788)
**Years of professional experience**	0.00392	0.00469	0.00400	0.00329	0.00443*
	(0.00302)	(0.00325)	(0.00303)	(0.00226)	(0.00244)
Bachelor	-0.0598	-0.0178	-0.0715	-0.109**	-0.0863*
	(0.0592)	(0.0696)	(0.0595)	(0.0439)	(0.0522)
Master	-0.0624	0.0766	-0.0630	0.0690	0.191
	(0.359)	(0.378)	(0.359)	(0.266)	(0.280)
Midwife		-0.0498			-0.118
		(0.163)			(0.122)
Physician		-0.151			-0.114
		(0.209)			(0.155)
Dentist		-0.0685			-0.131
		(0.208)			(0.155)
Other		-0.161			-0.170
		(0.172)			(0.127)
Nurse		-0.226			-0.210*
		(0.162)			(0.121)
Health promotor		-0.155			-0.156
		(0.209)			(0.156)
Nutritionist		0.0126			0.0129
		(0.198)			(0.147)
Pharmacist		0.0228			-0.0207
		(0.200)			(0.149)
Sanitarian		0.0272			-0.0622
		(0.176)			(0.131)
Hours spent working in PHC Facility per day		0.00355			0.00471
		(0.0339)			(0.0252)
Extra task as assigned		-0.00270			0.0290
		(0.0755)			(0.0562)
Have extra tasks (related COVID-19) but no specific task assigned		-0.113			-0.165**
		(0.0994)			(0.0746)
testing		0.0422			-0.149
		(0.181)			(0.137)
tracing		0.0514			0.0151
		(0.119)			(0.0899)
treatment		0.0884			-0.0201
		(0.208)			(0.157)
vaccination		-0.0748			-0.0845
		(0.0809)			(0.0611)
**Side hustles**		0.0813			0.0133
		(0.0710)			(0.0543)
**Caring for aging parents and/or under-five children**			0.0422		0.0322
			(0.0667)		(0.0520)
**Helped by domestic servants**			0.0883		0.00740
			(0.0604)		(0.0490)
**Work interference with personal life (WIPL)**				-0.128***	-0.123***
				(0.0313)	(0.0328)
**Personal life interference with work (PLIW)**				-0.253***	-0.254***
				(0.0372)	(0.0395)
**Work enhancement of personal life (WEPL)**				-0.0973***	-0.109***
				(0.0344)	(0.0357)
**Personal life enhancement of work (PLEW)**				-0.122***	-0.113***
				(0.0354)	(0.0371)
Constant	1.469***	1.555***	1.401***	3.817***	3.879***
	(0.115)	(0.313)	(0.125)	(0.174)	(0.284)
Observations	308	308	308	308	308
R-squared	0.013	0.058	0.024	0.467	0.496

Robust standard errors in parentheses

^***^ *p* < 0.01, ** *p* < 0.05, * *p* < 0.1

In the fourth model, we found that all work-life balance dimensions were significantly associated with stress among primary health workers. We found that both dimensions representing interference between work and personal life (WIPL and PLIW) were negatively associated with stress. A higher score of WIPL than PLIW indicated low interference between health workers' roles in their work and personal life, which is advantageous for work-life balance. Hence, this result showed that more work-life balance is likely associated with lower stress perceived by primary health workers. The fourth model also informed that a high enhancement of work on personal life (WEPL) and personal life on work (PLEW) was also negatively associated with stress. Those enhancements provide more work-life balance and lower perceived stress among primary health workers. Those work-life balance dimensions explained 46.7% of the variance.

Lastly, we included all variables in our fifth model. When all work and personal life variables were combined with all the work-life balance dimensions, the *R*2 was 49.6%. We found that the increasing years of professional experience were significantly associated with higher health workers' perceived stress. More senior primary health workers likely had higher perceived stress than their juniors. Bachelor's education degree and being a nurse were associated with lower perceived stress. Respondents with a bachelor's degree were likely to have less stress than those with vocational education. The nurse was the only health worker occupation significantly associated with lower stress.

Interestingly, the fifth model informed how the COVID-19-related work-life balance determined the stress level among primary health workers. In contrast with our second model, which showed that work conditions did not significantly associate with stress, our last model showed that work-condition related to COVID-19 handling was significantly associated with stress. Although extra tasks outside their primary job and side hustles did consistently not associate with stress in all regression models, extra tasks related to COVID-19 handling in our fifth model were significantly associated with stress levels. Using having no task-related COVID-19 as a reference, we found that having an additional specific task related to COVID-19 handling, which means only assigned to tracing, testing, treating, or vaccinating, was not associated with their stress. In contrast, primary health workers with multiple task-related COVID-19 were associated with having lower perceived stress levels.

## Discussion

At the beginning of the COVID-19 pandemic, health workers must deal with minimal information on COVID-19 as a new disease and its dynamic handling policies, which create anxiety and additional workload [4]. The increasing workload also increased role conflict between health workers' work and personal lives [11]. Health workers with complex adaptation skills toward these rapid changes are having risk for higher stress [18]. Our study was conducted in the third year of the pandemic and contributed to our understanding of how the pandemic impacted primary health workers' work-life balance and stress in the long years of the pandemic.

This study found that, during the pandemic, the personal life of primary health workers interferes with work life more than enhances it. Most of our respondents had elderly or under-five children as their dependents and did their domestic work independently without help from domestic servants. Those personal life conditions increased their anxiety in conducting their roles as health workers who must be exposed to COVID-19 workload [30]. They feared being transmitted by COVID-19 and infected their families while they must still organize domestic work under their increasing COVID-19 additional workload [11, 13, 30]. Hence, having much pressure from their personal life when conducting their job made their compliance in work life more difficult. When personal life interferes affects work, individuals may find it challenging to focus on their work and complete tasks efficiently, resulting in decreased job satisfaction associated with higher stress [20, 31, 32].

Primary health workers in Indonesia were on the frontline and took responsibility for COVID-19 tracing, testing, treatment, and vaccination. These additional COVID-19 workloads were also faced by most of the primary health workers in our study. Our study found that work life's interference and enhancement scores on personal life were equal even with a low score. It indicates that their work life during the pandemic brought both negative and positive spillovers in their personal lives, leading to a state of balance in which individuals can manage the demands of their work without experiencing significant negative impacts on their well-being. However, since the scores were relatively low, it can negatively impact individuals' well-being, which we measured as perceived stress in this study. Working conditions with excessive workloads during the pandemic resulted in fatigue and stress among health workers [8, 9]. Lack of fulfillment due to the absence of activities that enhance their personal lives would lead to poor mental health [33, 34]. Primary health workers may have less energy, motivation, and focus on devoting themselves to their work, leading to lower work performance and more conflicts in their personal life [20, 35, 36]. Prioritizing activities that enhance personal lives and striving for a healthy work-life balance is crucial to avoid those negative consequences.

Most importantly, our study found that the stress level among our respondents was moderate, but the work-life interfered with health workers' personal life. These findings verify the work-life imbalance among primary healthcare workers during the third year of the COVID-19 pandemic, associated with higher perceived stress. As the health workers who became the frontline of the testing-tracing-treatment strategy and the COVID-19 mass vaccination, primary health workers were assigned more extra work during the pandemic. This study found that extra tasks outside their primary job unrelated to COVID-19 handling did not associate with higher stress. In contrast, the multiple extra tasks related to the COVID-19 control measures were associated with lower stress among primary health workers. This finding differs from other studies, which reported that the increased workload due to the pandemic increased the health workers' stress levels [16, 18]. Considering the data collection period of our study conducted in mid-2022, in the third year of the COVID-19 pandemic, primary healthcare workers may have better perceptions of COVID-19 and its risks in the workplace. Primary healthcare workers with better perceptions of COVID-19 and attitudes towards responsibility for COVID-19 risk at work reportedly are more committed to taking more roles in COVID-19 handling [37].

We also found that multiple tasks related to COVID-19 are likely more fruitful for primary health workers rather than only working monotonously on COVID-19 handling. It is probably related to workplace conditions. Workplace factors, including personal protective equipment (PPE) availability, staff training, and provision of mental health support, were reportedly associated with mental health during the COVID-19 pandemic [38]. Since the pandemic began, primary healthcare workers in Indonesia must struggle with additional workloads with unfavorable workplace factors [39–41]. While primary healthcare workers are still conducting their original duties, their additional workloads related to COVID-19 handling are also evolving based on the development of COVID-19 [39]. In the first year of the pandemic, the additional workload was only related to contact tracing and testing [39, 41]. In the second year of the pandemic, the vaccination workload was added and continued with a more vaccinated target population in the following years [40]. Hence, in the third year of the pandemic, health workers with multiple workloads likely consider the COVID-19 task routine work and develop coping strategies. In addition, multiple tasks provide more variation and challenge for health workers, reducing boredom and increasing their performance [42, 43].

Health workers' resilience in managing stress may vary between individuals since it is a complex construct influenced by various factors [29, 38, 44]. Our study found that gender, age, and marital status of primary health workers did not determine stress levels, but their years of experience are likely related to higher perceived stress. Our data shows that primary healthcare workers with shorter work years are less stressed. Our findings could be explained by the ability of workers with fewer years of experience to adjust to rapid environmental change, a remarkably rapid change caused by the pandemic [41, 42]. It differs from a recent study that discovered that senior health workers could improve coping skills during stressful situations [19]. Nevertheless, it is consistent with a study that indicated that work stress rose with age due to older workers' work, health, and socioeconomic susceptibility [40].

At the same time, our findings show that being a nurse was the only health profession significantly associated with lower perceived stress, which could be explained by the psychological changes experienced by this profession. A study reported that female nurses in Indonesia, which also found less stress in this study, have experienced psychological changes during the pandemic, from feeling hopeless at the beginning of the pandemic to finding pride in saving patient lives [45]. Since stress can arise from diverse sources and constantly changing [44, 46], stress management among primary health workers has become an ongoing process during the evolving years of the pandemic.

Our respondents with a bachelor's level of education have less stress than the vocational level. It could be understood since the vocational level health workers in Indonesia's primary healthcare facilities are the frontline in the field while the bachelor is commonly placed in managerial positions that do not frequently meet patients. Frontline health workers working in high-risk COVID-19 transmission developed a perceived threat of COVID-19, increasing their burnout and resulting in higher stress levels [4]. While due to the choice of cross-sectional design, our study has limitations in being unable to identify the duration of work-related stress levels.

Furthermore, we found that work-life balance is associated with primary healthcare workers' perceived stress levels. This work balance is manifested in two different conditions. First, work and personal life interference are associated with higher primary health workers' perceived stress. This condition indicated any conflict between health workers' personal and work lives, in this study, likely more contributed by work-life condition. Other studies fully support this finding, which found that role conflicts among health workers reportedly increase their stress levels [11, 18]. Health workers are vulnerable to mental ill health because of their overwhelming workload and high demand for personal accomplishment during the COVID-19 pandemic [47]. Second, work and personal life enhancement are associated with lower perceived stress. Social supports received by primary health workers reportedly decrease their stress [11]. The personal relationships of health workers with their family members and spouse enhanced their work-life balance during the pandemic [17]. Our study strengthened it, which found that personal life interference with work life is the lowest among other work-life balance dimensions and is associated with lower stress. Work support from their working circles significantly minimizes the interference between work and personal life [35].

## Conclusion

During the third year of the COVID-19 pandemic, primary health workers' work life interferes with their personal life more than enhances them. At the same time, their personal life has a lower interference and higher enhancement on their work life. This study found that all work-life balance dimensions are associated with perceived stress among primary health workers. Low interference of work life in personal life and vice versa is associated with low-stress levels, while high enhancement of work life in personal life and vice versa are associated with low-stress levels. This perceived stress is also associated with primary health workers' years of experience, occupation, education level, and multiple tasks related to COVID-19 handling assigned. Primary health workers with longer experience years likely have a higher stress level, while those with bachelor's degrees are likely to have lower stress than those with vocational education. Being a nurse is likely associated with lower perceived stress. Our findings also suggest that compared to health workers with no task-related COVID-19, having an additional specific task related to COVID-19 handling does not associate with their stress level. However, having multiple task-related COVID-19 during the third year of the pandemic is associated with lower perceived stress levels.

## Supplementary Information

**Additional file 1: Supplementary Table 1.** Correlations among variables.

## Data Availability

The datasets used and/or analyzed during the current study are available from the corresponding author upon reasonable request.
